# The reform of the essential medicines system in China: a comprehensive approach to universal coverage

**DOI:** 10.7189/jogh.03.010303

**Published:** 2013-06

**Authors:** Sarah L. Barber, Baobin Huang, Budiono Santoso, Richard Laing, Valerie Paris, Chunfu Wu

**Affiliations:** 1 World Health Organization, China; 2 WHO Western Pacific Region, Manila; 3 World Health Organization Geneva, Switzerland; 4 Organisation for Economic Co–operation and Development (OECD) Paris, France; 5 Shenyang Pharmaceutical University, Shenyang, China

In OECD countries, medicines spending accounts for approximately 17% of total health spending or 1.5% of gross domestic product (GDP) [1]. New technologies and pharmaceuticals have been important contributors to rising health care costs. At the same time, patients may not have access to cost–effective medicines because of lack of health insurance coverage, limited insurance benefits, high medicines prices, physician prescribing choices, or differences between available essential medicines and consumer demand [2]. With the exception of a few countries [3], however, the approach to reform tends to be piecemeal rather than comprehensive.

With the goal of universal health care coverage by 2020, the Government of China has implemented comprehensive health care reforms nationwide [4]. Between 2009 and 2011, the reforms focused on increasing access to essential medicines as well as expanding health insurance, strengthening the primary care system, financing public health, and reforming public hospitals [5]. By 2011, government investments in health reform reached Yuan 1.13 trillion (US$ 174 billion, at Yuan 6.5 per US$) [6]. Total health expenditures increased from 3.5% to 5.0% of GDP between 1995 and 2010 – amounting to an increase from US$ 21 to US$ 220 per capita [7].

The reform of the essential medicines system is a major focus of the national reform agenda. Spending on medicines accounted for 41.9% of total health expenditures in 2010, or 2.1% of GDP (**Figure 1**) [7]. In this paper, we review existing literature, published government documents about the essential medicines reform in China, and international literature on essential medicines and health care reform internationally. The paper first presents economic and demographic trends to explain rapid increases in medicines consumption across China. The 2009 health care reform is discussed, in terms of each component’s linkage with medicines reforms. We discuss in detail the reform of the essential medicines system, including the Essential Medicines List (EML), procurement, pricing, financing, and quality. We conclude that China’s comprehensive approach in reforming its essential medicines system could be a model for other countries that strive to ensure access to medicines while also controlling costs.

**Figure 1:**
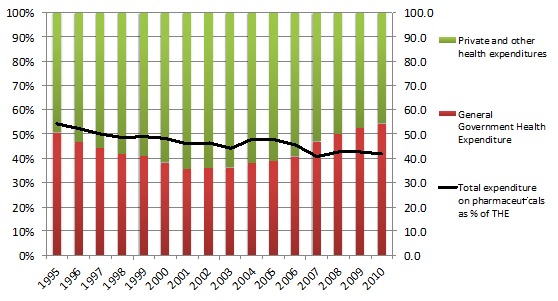
Private and general government health expenditures, and percent of total health spending devoted to pharmaceuticals, 1995–2010. Source: ref. [7].

## ECONOMIC AND DEMOGRAPHIC TRENDS DRIVING ESSENTIAL MEDICINES CONSUMPTION

**Figure 2:**
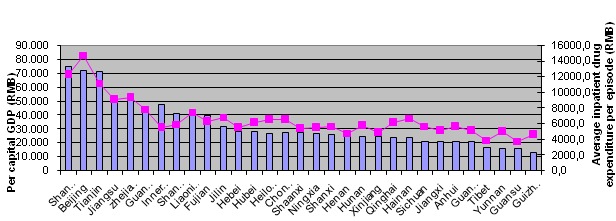
Per capita GDP and average medicines expenditure per inpatient visit, by region 2010.Source: ref. [10].

Economic and demographic factors have resulted in increased demand for essential medicines. GDP growth is projected at 7.5% in 2012 [8]. Positive economic growth increases the government’s ability to invest in reforms, and household disposable income has also increased between 2008 and 2011 [9]. Strong correlations exist between provincial wealth and spending on medicines, as illustrated by the average medicine spending per inpatient visit by province (**Figure 2**).

**Figure 3:**
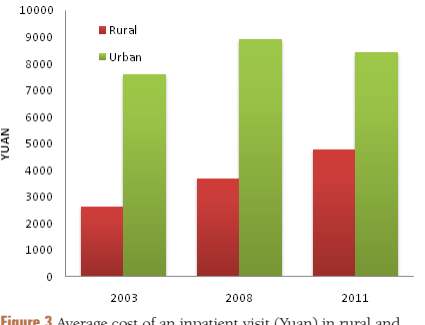
Average cost of an inpatient visit (Yuan) in rural and urban areas, in 2003, 2008, and 2011.Source: ref. [11].

Urban residents tend to have higher disposable income, and access to a greater supply of medical products and services. In 2011, average inpatient fees in urban areas were US$ 1340 – nearly twice as high compared with rural areas (US$ 760) (**Figure 3**) [9,11]. By the end of 2011, urban residents increased to 690.8 million people, or 51.3% of the population [10]. With rapid urbanization, increasing numbers of people are changing their lifestyles in such a way that may promote chronic diseases – such as decreasing their physical activity. Moreover, by 2030, people 65 years and older will account for at least 20% of the population, representing 240 million people [12]. Economic growth, urbanization, and population aging together are expected to contribute to a 40% increase in the non–communicable disease burden by 2030[12]. At the same time, less than half of patients of with hypertension and diabetes are diagnosed and even fewer receive effective treatment [13], suggesting a large and growing need for essential medicines for the treatment of long–term chronic conditions.

Medicines to treat many non–communicable conditions are expected to become more accessible. By 2015, 40% of the current patent–protected products will be developed as generics as the patents expire [14]. This will lead to the increased use of these medicines as generics in China, where they will become more cost–effective for inclusion under public health care benefit packages – and more accessible for patients who pay out–of–pocket.

## THE REFORM OF THE ESSENTIAL MEDICINES SYSTEM

The national health care reform announced in April 2009focuses on strengthening insurance, public health services, service delivery, public hospitals, and essential medicines. **Table 1** summarizes the policy directions for the main reform components [4,5,15,16,18], and the implications for medicines affordability and access. **Table 2** presents a summary of the essential medicines reform.

**Table 1:** National health care reform in China: The impact of health care reforms on access to and utilization of essential medicines*

Area of reform	Impact on essential medicines
**Social security and insurance:**	
	Large–scale increase in the number of people covered under formal insurance programs, from 294 million in 2003 to 1.28 billion by 2011 (21.0% to 93.0% coverage).
	Insurance reimbursement lists are required to incorporate the medicines on the Essential medicines lists (EML) at central and provincial levels, at higher reimbursement rates compared with medicines not on the EML.
	Inpatient insurance reimbursement rates rose steadily, averaging 46.9% in 2011, including medicines and service fees.
	Per capita premiums for basic health insurance programs to increase to 360 Yuan (US$ 57) per person by 2015, from about US$ 32 in 2010.
**Service delivery:**	
	Reconstruction of the primary care system, including some 2200 county hospitals and 33 000 urban and rural primary care facilities.
	In government–run primary care facilities, comprehensive financing reform under way to replace revenue from medicines sales to fund operational costs, through increased insurance and government subsidies.
	Greater emphasis on quality, through clinical treatment guidelines, hospital formularies, and prescription monitoring systems.
	By 2015, the government aims to achieve 90% of outpatient utilization at county level or below.
**Public health:**	Ten categories of basic public health services have been implemented, through a per capita subsidy (25 Yuan) to primary care facilities. The subsidy is targeted to increase to 40 Yuan by 2015.
	The public health subsidy replaces to a large extent the revenue lost through the zero mark–up policy for essential medicines, and covers a large share of operational costs at township hospitals, village clinics, and community health services centers.
	Eight categories of major public health services, including expanded access to millions for Hepatitis B vaccines, cervical and breast cancer screening.
**Public hospital reform on pilot basis:**	17 municipalities and 37 provincial cities were designated to undertake hospital reform on a pilot basis, to reduce the reliance on medicine sales as a major source of revenues. The main activities include provider payment reform (mainly DRGs and case based payments) and clinical pathways, setting fixed prescription fees, and setting up independent pharmaceutical distribution networks.
	In 300 county hospitals in 2012, it is proposed to eliminate completely the medicines bonus policy, whereby staff are rewarded for over–prescription.

*Data sources refs. [4], [5], [15-17].

**Table 2:** National Health Care Reform in China: Summary of activities under the reform of the essential medicines system: 2009–2011 and directions for 2012*

Area of reform	Major activity	Major impact
**Essential medicines lists**	Essential medicines list (EML) for primary level care issued at central and provincial levels. Revisions to be issued in 2012.	Essential medicines available at primary care facilities at cost.
**Insurance reimbursement lists**	Insurance reimbursement lists were issued at central and provincial levels, which include the medicines on the EML, at higher reimbursement rates.	Inpatients are reimbursed for essential medicines at higher rates than non–essential medicines.
**Procurement**	Centralized procurement and bidding platforms implemented at provincial levels, including online purchasing. Efforts are made to reduce the number of distributors and mark–ups in the distribution chain. The two–envelope system is encouraged, to ensure minimum quality standards under the tendering system prior to consideration of the commercial bid.	Prices for essential medicines have been reduced primarily through greater efficiencies.
**Pricing**	Systems have been established for setting and adjusting guiding retail prices for essential medicines.	Through release of pricing data, greater price transparency is possible.
**Financing**	Essential medicines are provided at cost (zero profit mark–up) at all government–run primary care facilities in urban and rural areas. Comprehensive financing for primary level facilities to replace revenue from medicines sales, and reform of prices. Zero–mark up will be expanded to village clinics, non–government run primary care facilities, and pilot county hospitals.	Prescribing and physician remuneration/facility operational costs have been delinked at many primary care facilities, thus reducing the incentives for over–prescription.
**Quality**	More intensive efforts to improve quality standards for 307 drugs on the national essential medicines list, including routine sampling and testing, electronic bar codes required on packages for monitoring. Strengthened systems for adverse drug effects.	Consumers have greater protection through quality standards, and more confidence in the quality of medicines.
**Rational medicines use**	Clinical treatment guidelines and formularies of essential medicines formulated and issued, and prescription monitoring systems put into place.	Increased knowledge of rational medicines use.

*Data sources: refs. [4], [5], [15-17].

### Essential medicines lists (EML)

The Ministry of Health first published its EML in 1982. By 2004, the EML (for primary and secondary levels) included 2033 products, including 1260 Chinese herbal preparations and 773 chemical and biological medicines products. To increase their availability, essential medicines were subject to price controls. However, this had the perverse effect of reducing financial incentives for their production, resulting in the lower availability of highly cost–effective preparations [17]. Until 2009, the EML was not used in financing and insurance reimbursement schemes. With the expansion of coverage and increase in reimbursement levels, the EML has become more important in defining benefits under the insurance schemes.

After health care reform was announced in 2009, the government issued a revision of the first part of the EML for primary care. This EML is currently composed of 307 medicines, including 102 traditional medicines. It is expected that the second part of the EML for secondary hospitals will be issued at a later date. Following the completion of the central list, each province and municipality prepares its own EML. A study of 22 provincial EMLs reported that wealthier provinces added on average 236 medicines to the list, and less prosperous provinces added 107 products [19].

The selection criteria include clinical need, safety and efficacy, price, availability from suppliers, clinical treatment guidelines, and appropriateness for use at primary care level. The selection process for the EMLs and the medicines reimbursement lists, however, is not yet standardized across provinces. The experts participating in the selection may not have access to up–to–date and independent evidence to make informed decisions about cost–effectiveness, safety, and efficacy [19]. In addition, the systems for managing conflict of interest are not yet in place. The process relies primarily on expert opinion rather than objective evidence – which lacks credibility and acceptability among other experts and health professionals that are not involved.

It is tempting to compare the Chinese EML for primary level with the WHO 2011 Model List. However, it is important to recall that the lists serve different purposes. The WHO Model List covers both primary and hospital conditions and addresses some diseases that are not found in China. It includes anesthetic agents, cancer therapies and other medicines that would only be used in hospitals. The WHO Model List does not include traditional medicines.

### Health insurance reimbursement lists

Following the issuance of the EML in 2009, the Ministry of Human Resources and Social Security (MoHRSS) conducted a selection process to issue the central medicine reimbursement list for the urban employee and urban resident insurance programs. The 2009 list includes 2151 products (1164 western and 987 traditional medicines), categorized into List A and List B. All medicines in the EML are in List A, which are reimbursed at higher rates compared with non–essential medicines. The MoHRSS plans to revise the central reimbursement list every four years. Central guidelines indicate that up to 15% of medicines on List B can be adjusted by provinces to meet their own health needs.

While the situation is evolving rapidly, the rural and urban insurance programs focus on the coverage of inpatient care, subject to deductibles and caps. A deductible is the amount paid out–of–pocket by the patient before any insurance payments are paid. For the urban programs, wide variations exist across municipalities, and most municipalities reimburse for primary level outpatient services and medicines depending on their economic capacity. For example, a 2009 study reported that diabetes patients in Huangshui, Hubei, were not subject to deductibles, and were reimbursed at 75% until reaching an annual cap of US$ 267. In contrast, diabetes patients in Shantou, Guangdong, faced a deductible of US$ 158, a reimbursement rate of 50%, and a cap of US$ 952 [20].

Unusually in China, the EML serves as a minimum list of basic medicines, and the insurance reimbursement lists tend to be much longer compared with the EML. The question is whether funding to “non–essential medicines” diverts resources from other more cost–effective care. Public health benefit and cost–effectiveness could be used in both the selection of the EML and insurance reimbursement lists. Under ideal conditions, the basis for the development of the EML and the insurance reimbursement lists should be evidence–based clinical practice guidelines. Currently, existing clinical practice guidelines and medicines formularies are not widely and consistently used in practice.

### Procurement

Before the reforms, procurement occurred at facility levels; there were many small–scale fragmented distribution systems and large numbers of wholesalers and distributors that contributed to higher mark–ups. The reforms aimed to consolidate the numbers of firms and agencies involved in drug procurement and distribution, reduce costs, and monitor more closely the performance of suppliers. By the end of 2010, government–led bidding platforms were established in all regions, and the majority of counties implemented online purchasing. In general, provincial procurement has promoted greater efficiencies in management. These efficiencies combined with higher volume purchasing have resulted in reductions in medicines prices. The government reported that the price of essential medicines dropped on average by 16.9% between 2009 and 2011 [21]. Independent small–scale studies demonstrated even larger reductions in medicines prices [22]. Yang et al report declines in the average cost per prescription from 45 to 27 Yuan in Hubei province [23].

The central government advocates for the use of the “two–envelope” tendering system, which is under way in some provinces. Under this system, suppliers submit two sets of documents in the bidding process. The first set of documents demonstrates the supplier’s compliance with quality and performance standards. For those suppliers that meet the quality standards, the commercial bid is evaluated. However, at present, the procurement process and logistics capacity across the provinces are not uniform; and the systems, specifications, and criteria vary.

Even though large numbers of firms participate in the procurement process, the bidding tends to result in one firm winning the tender for one product. Under most of the current provincial models, the authorities place the strongest emphasis on obtaining the lowest possible price, and pay less attention to medicines quality and reliance supplier performance. This has resulted in price wars among manufacturers, whereby firms submit commercial bids that are below cost. Generally, over–reliance on single–source suppliers may carry risks in decreased competition for certain products. Decreased competition may result in fewer firms registering production and effectively leaving the market. Thus, the system of relying on single–source suppliers could be reevaluated, to ensure that there are sufficient suppliers in the market to ensure competition and choice.

In some regions, medicines on the provincial EMLs are not easily procured. For example, in Fujian province, 42 products on the EML could not be easily procured. The main reasons include lack of supplier, sole source manufacturers, and firms’ non–acceptance of the tendering price. In all regions, procurement authorities conduct negotiations among sole source suppliers for products on the EML that are not easily procured.

### Pricing

The Provincial and National Development Reform Commissions (NDRC) regulate prices for approximately 2700 items, among the estimated 11 000 medicinal products marketed in China. Medicines on the central and provincial EMLs and reimbursement lists are subject to price controls, whereby the NDRC establishes maximum retail price ceilings used for procurement and dispensing. Multiple price changes have been introduced for essential medicines since the mid–1990s.

**Figure:**
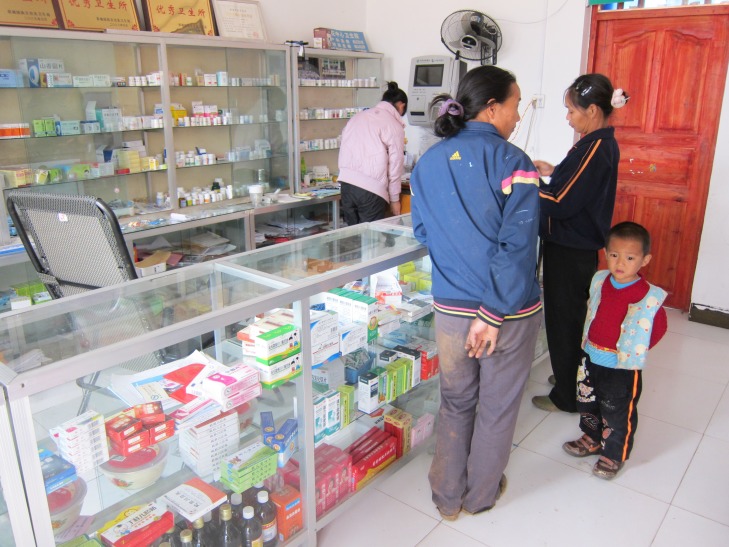
Photo: Courtesy of Kit Yee Chan, personal collection

For most essential medicines, NDRC sets the maximum retail prices used for procurement; the actual retail price is determined through competitive bidding by regional authorities and mark–ups at distribution and facility levels. The NDRC collects and verifies data submitted by firms and wholesalers, retail prices from trade associations, provincial procurement prices, and cost studies by manufacturers. There is some variation by type of product, whereby patented medicines, for example, rely on pricing by pharmaceutical companies. In addition, firms that invest in quality improvements and comply with international quality standards – as indicated by an international GMP certification, for example – are eligible to negotiate preferential prices.

The pharmaceutical pricing policy in China contrasts with policies adopted in other countries in several ways. First, it relies on cost–plus accounting methods for pricing rather than commonly used methods used in other countries, such as international benchmarking, internal reference pricing, or pharmacoeconomic analysis. Second, it regulates prices for both branded single–source and generic multi–source products. Many countries rely on market controls for generic multi–source products that can be procured through tendering where sufficient market competition exists. Third, pricing information is generally not made available to the public, although retail medicines prices are available at public health institutions. Finally, a 17% Value Added Tax (VAT) is applied to all medicines, which is relatively high in comparison with OECD countries [24].

In the next 5–year plan, the government intends to strengthen price transparency and disclosure. This is an important step given that greater price transparency at all stages of the medicine supply chain could reduce extreme price variations. Strategies to increase price transparency has been successful in countries such as Brazil, for example, where public procurement authorities can increase their bargaining power by knowing whether prices are competitive [25].

### Comprehensive reform of primary care facilities

**Figure 4:**
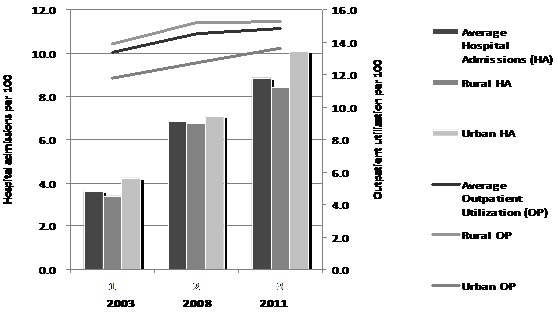
Increases in outpatient utilization (lines) and hospital admissions (bars): 2003, 2008, and 2011. Source: ref. [11].

Great efforts have been made to encourage utilization of essential medicines and services at primary levels. Making essential medicines available at procurement cost (the zero mark–up policy) was one of several strategies to strengthen the primary care system, alongside infrastructure investments and deployment of qualified health workers [26]. However, the increases in outpatient utilization during the reform period are modest relative to the large increases in inpatient admissions reported since 2003 (**Figure 4**).

At present, there is no separation between prescribing and dispensing, and both are done within the same public health facilities. To reduce the cost of medicines, they are sold at cost in all public primary level facilities. This policy has now been implemented in all government–run primary level facilities. The share of government subsidies to support operational costs at grassroots facilities has increased steadily to compensate for the reduction in revenues from medicines sales. Local governments are mainly responsible for replacing the revenues lost through the zero mark–up policy. In some cases, provincial or central government support is weak, thus placing high financial pressures on local governments, particularly in poor and near poor regions. Insufficient government subsidies may result in shortages in routine operational activities, weak ability to maintain the essential medicine system, or sales of diagnostics, technologies, or other revenue generation methods to cover basic operational costs.

Local governments determine whether to implement a companion policy, to separate revenues and expenditures in government–run primary health institutions. In this model, medicines sales revenues are returned to the county finance department. The institutions are then allocated budgets based on actual costs. In many facilities, however, partial or no separation of revenues and expenditures occurs. Thus, the allocation of government subsidies continues to be based on prescription volumes and represents only the lost profit mark–up. In this case, associations remain between health provider remuneration and volumes of services, diagnostics, and imaging, and the incentives for over–prescription are the same as before the reform.

### Ensuring medicines quality

Under the reform targets for medicines quality, the government put into place stronger production standards, safety regulations, and post–marketing surveillance. The key achievement was the implementation in 2011 of the revised Good Manufacturing (GMP) standards, which do not differ in any critical ways from the WHO GMP standards. It is anticipated that GMP implementation will result in consolidation across some 4600 manufacturers registered to produce finished pharmaceutical products (FPPs) and/or active pharmaceutical ingredients (APIs) [27].

Since 2000, a series of government policies were put into place to promote higher quality standards [28]. A persistent problem has been the quality standards for traditional medicines [29]. In 2011, the government announced strengthened regulations to promote quality in the production, sales and use of prepared slices of Chinese crude medicine. Since 2009, all medicines on the essential medicines list (EML) are required to undergo quality sampling and testing at provincial level annually, and at central level every three years. Substantial numbers of TCM injectables are included on the central and provincial EMLs, and these products pose the highest safety risks. Thus, greater attention to the highest risk products is warranted.

The majority of the medicines on the list are generics. At present, generic products can be used as comparators for generic medicine approvals, where an originator product is not available. This may lead to lack of inter–changeability across generic products, and between generics and the originator products.

## DISCUSSION AND CONCLUSIONS

The implementation of the WHO essential medicines concept is intended to be flexible and adaptable to many situations. The Chinese reforms were grounded in the WHO concept of Essential Medicines established in 1975 by the World Health Assembly, which aims to ensure medicines access and affordability, rational use, and quality and safety[30].Policies have been implemented to promote the availability of essential medicines of assured quality at affordable prices. The essential medicines reform is comprehensive and includes systematic selection of essential medicines, centralized procurement and tendering at provincial levels, pricing policies, provision of essential medicines at cost in primary level facilities (zero mark–up), and stronger quality and safety standards. A great deal of progress has been achieved within a short timeframe. The health reform implementation plan for 2011–2015 has set forth activities to consolidate and expand on these gains [18]. This systematic and comprehensive approach applied in China serves as a useful model for other countries in reforming its health care system.

A few challenges remain. It is essential to accelerate the introduction of a scientifically robust system of inter–changeability of generics, which would pave the way towards comprehensive policies to promote the use of generic medicines. The development of objective and evidence–based guidelines remains an important yet neglected part of the reform. Under ideal conditions, the basis for the development of the EML and the insurance reimbursement lists should be evidence–based clinical practice guidelines. Information required for developing evidence–based guidelines includes current utilization patterns for services and medicines. Practice guidelines should start with common conditions of public health significance and high usage medicine products. Reimbursement lists should utilize the same information, and financial incentives could be provided to promote utilization at primary levels and essential medicines. Where guidelines are used as the basis of public funding, it is important that the process of guideline development is consistent with international standards.

In terms of procurement, provinces will need to adopt stronger and more uniform criteria to accurately capture product quality and firm performance in contract delivery. Consideration could be given to different types of price setting mechanisms to ensure that prices are set with some reference to their therapeutic value. In addition, price controls need to address the mark–ups and VAT, and also the volumes prescribed. Consumer education is an essential part of successful essential medicines policies, to ensure that consumers understand and trust essential medicines. Patient and consumer education should be incorporated into the national policy framework.

Under the current system, increased use of essential medicines relies on the shift in patient care seeking behaviors from hospitals to primary care facilities. This shift will require improving quality of care and human resources at the primary level, and aligning the incentives in the insurance systems to promote referral systems. Piloting alternative ways of paying health care providers is under way across China, and an expansion of these pilots is envisioned.

It is quite early in the reform process to expect major changes in medicines spending. However, this paper illustrates a complex sequencing of events, whereby the institutional structures and policies are put into place first, complementary reforms including insurance expansion are under way at the same time, and comprehensive monitoring systems are established to enable adjustments along the way.

In China, a comprehensive approach to implementing the essential medicines concept was required given that medicines sales were the basis for operating costs for health facilities. The reform in essential medicines is linked closely with the success of improving quality of primary care facilities and implementing public hospital reforms. The comprehensive approach to implementing the essential medicines concept in China is useful for other countries that seek to achieve universal coverage, and ensure medicines access and affordability, rational use, and quality and safety.

### What we already know about the topic concerned

The problems of access and affordability in China’s health system have been well documented [31]. The ambitious design and objectives of national health care reforms in 2009 were publicized [4,5]. Existing studies and reviews have documented trends in access and financial protection, with a focus on insurance reforms, access, and the increase in spending on health [9]. It has been documented that a large share of total health spending is dedicated to pharmaceuticals, amounting to 2.1% of GDP [7]. Prior studies of essential medicines in China have focused on narrow aspects such as production quality [28] and the selection process for the essential medicines list [19].

### What new knowledge the manuscript contributes

This manuscript provides a comprehensive description of the large–scale essential medicines reform under way in China. To the authors’ knowledge, no prior detailed description of activities under this reform component has been published previously. Given that medicines have long been used to fund operational costs for health facilities, reform of the essential medicines system can have a far–reaching impact on access and quality of health services. The comprehensive approach is an excellent example of adaptation of the WHO Essential Medicines Concept, which can be informative for other countries.
